# Effect of aromatherapy with *Matricaria chamomilla* in pain and anxiety management in hospital settings: A scoping review protocol

**DOI:** 10.1371/journal.pone.0339953

**Published:** 2026-01-05

**Authors:** Maria Améllia Lopes Cabral, Karena Cristina da Silva Leal, José Joandson de Souza dos Santos, Fernanda de Castro Teixeira, Ana Beatriz Santos Trindade, Auanna Beatriz Sarmento de Araújo Miranda, Lara Dantas de Rubim Costa, Daniele Vieira Dantas, Rodrigo Assis Neves Dantas

**Affiliations:** 1 Department of Nursing, Federal University of Rio Grande do Norte, Natal, Rio Grande do Norte, Brazil; 2 Heart Hospital, Natal, Rio Grande do Norte, Brazil; University of Toledo College of Medicine: The University of Toledo College of Medicine and Life Sciences, UNITED STATES OF AMERICA

## Abstract

**Introduction:**

Pain and anxiety are underestimated complications in hospitalized patients, negatively impacting their well-being and physiological functioning. Integrative and Complementary Health Practices (ICHP) emerge as alternatives or complements to standard treatments, with chamomile aromatherapy highlighted for its sedative, anxiolytic, and analgesic properties.

**Objective:**

This review aims to map the existing knowledge regarding the use of aromatherapy with chamomile essential oil in the management of pain and anxiety within hospital environments.

**Method:**

This study is a scoping review that adheres to the Joanna Briggs Institute (JBI) guidelines and the PRISMA-ScR checklist. A comprehensive search will be conducted across ten data sources for this review: Medical Literature Analysis and Retrieval System Online (MEDLine/PubMed), Cumulative Index to Nursing and Allied Health Literature (CINAHL), Web of Science, LILACS, Scielo, Cochrane Library, Scopus, ScienceDirect, Wiley Online Library, and Virtual Health Library (VHL). The selection of studies will include full-text articles, dissertations, theses, ministerial ordinances, and guidelines, while abstracts, letters to the editor, opinion articles, off-topic studies, and duplicates will be excluded.Results will be presented through a PRISMA-ScR flowchart and tables, employing the PAGER methodology to enhance reporting quality.

**Conclusion:**

Aromatherapy with chamomile essential oil shows promising results for managing pain and anxiety in hospital settings. It demonstrates mild analgesic effects and consistent anxiety reduction, especially when administered via inhalation. This review aims to systematize existing knowledge, identify gaps, and provide theoretical and practical support for its safe clinical application, thereby strengthening integrative practices and promoting more humanized care.

## Introduction

Pain and anxiety are highly prevalent and frequently underestimated complications in hospitalized patients. The International Association for the Study of Pain (IASP) defines pain as an unpleasant sensory and emotional experience associated with actual or potential tissue damage [1,2]. Several barriers impede an effective approach to pain and anxiety, including adverse drug effects, patient hesitation regarding medication use−especially opioids−and a lack of health coverage, as well as adequate human and structural resources [2]. Concurrently, anxiety, which can even be pain-induced, is a prevalent symptom that, if not properly addressed, can lead to other systemic complications such as palpitations, tachypnea, nausea, depression, dyspnea, and recurrent pain, thereby creating a vicious cycle. This underscores the urgent need for safe and low-risk complementary approaches to alleviate patient suffering [3].

In this context, according to the Pan American Health Organization (PAHO) and the World Health Organization (WHO), Traditional, Complementary, and Integrative Medicines (TCIM) are defined as a set of practices based on theories, experiences, and beliefs from various cultures that are not part of conventional medicine [4]. Generally, these practices are utilized with a holistic perspective of the individual, aiming for health promotion, prevention, and recovery [5,6]. Furthermore, 170 out of 194 WHO member states recognize the use of traditional and complementary medicine, and 50% of these members have a national policy on traditional and complementary medicine [7].

In Brazil, the legitimization and institutionalization of these healthcare approaches began in the 1980s, particularly after the creation of the Brazilian Unified Health System (SUS). In 2006, through Ministerial Ordinance MS/GM No. 971, the National Policy on Integrative and Complementary Practices (PNPIC in Portuguese) was consolidated to officially institutionalize Integrative and Complementary Health Practices (ICHP), which are referred to as TCIM by the WHO, and provide a perspective directed toward continuous, humanized, and comprehensive health care [5]. The PNPIC was established to address the needs of the Brazilian population, the demand for the standardization and harmonization of these practices within the public health network, and the guidance from the WHO. Among its objectives, it proposes to “contribute to increasing the system’s problem-solving capacity and expanding access to integrative and complementary practices, ensuring quality, efficacy, efficiency, and safety in their use”. This definition can facilitate a holistic approach to health [6,8,9].

Among the diverse therapeutic resources within ICHP, examples include biodance, chromotherapy, chiropractic, music therapy, yoga, floral therapy, and aromatherapy, among others. These are used globally to manage pain, depression, anxiety, chronic disease symptoms, sleep disorders, and more [6,10]. Aromatherapy is characterized as a secular therapeutic practice that utilizes the properties of essential oils to restore the body’s homeostasis and harmony across physical, mental, well-being, and hygienic aspects. It is a multiprofessional practice adopted by various healthcare professionals such as nurses, psychologists, physical therapists, and physicians, among others, and is employed in different sectors of healthcare to complementarily assist in re-establishing an individual’s physical and/or emotional balance [5,8].

In this context, nursing can strengthen its evidence base, integrate complementary therapies into practice, and contribute to interdisciplinary dialogue in the field of TCIM/ICHP. With the increasing use of these therapies, nursing can play a crucial role in investigating their safety and efficacy, expanding patient-centered care, and fostering scientific collaboration among disciplines [6]. Among the substances commonly used in aromatherapy, chamomile (*Matricaria chamomilla*) stands out for its long history of traditional use. It has commonly been used as a mild sedative to induce neurological relaxation and reduce anxiety, as well as to treat insomnia through the inhalation of vaporized essential oils derived from its flowers. Furthermore, no significant side effects of chamomile use were reported in the studies included in a recent systematic review, indicating its safety for these patients [11–13].

Moreover, sources highlight chamomile’s efficacy in pain relief across various conditions. A recent systematic study identified chamomile as an effective treatment for primary dysmenorrhea, reported as more effective than placebo and NSAIDs (non-steroidal anti-inflammatory drugs) in some cases. Chamomile also proved effective in reducing pain associated with oral mucositis in cancer patients, and evidence suggests its potential in relieving postoperative pain and promoting wound healing, owing to its ability to reduce nociception and act as an anti-inflammatory agent [11–14].

The high prevalence of anxiety and other physical and psychological complications in hospitalized patients underscores the urgent need for effective interventions. Conventional treatments are accompanied by significant side effects, which drives the search for safe and low-risk complementary methods. Chamomile presents itself as a promising alternative, with growing evidence of its efficacy in mitigating various complications [3,11,15]. The ability of aromatherapy with chamomile to act as a non-pharmacological and safe measure suggests its potential for incorporation into routine care, alleviating patient suffering.

However, there remains a gap in the standardization of chamomile’s forms, doses, and durations of use, and few studies provide a comprehensive overview of its applicability and implementation as a complementary therapy in healthcare. Therefore, this scoping review is justified by the need to answer these questions and to synthesize existing knowledge on the use of aromatherapy with *Matricaria chamomilla* essential oil for managing pain and anxiety in hospitalized patients—a topic of clear clinical value and significance.

This review will contribute to evidence-based decision-making and practice. Furthermore, it will help guide future clinical research toward the standardization and safety of this intervention, which is notable for being low-cost, easy to apply, and feasible for implementation by various professionals, including nurses. The aim is to map the knowledge regarding the use of aromatherapy with chamomile essential oil in the management of pain and anxiety in hospital settings.

## Methods

This review protocol will be conducted in accordance with the guidelines formulated by the Joanna Briggs Institute (JBI) for scoping reviews and will be reported based on Preferred Reporting Items for Systematic Review and Meta-Analysis Protocols (PRISMA-P) 2015 checklist as detailed in S4 File [16,17].

This study was pre-registered on the Open Science Framework (OSF) public platform prior to commencing the formal search (registration link: https://osf.io/xrycm/?view_only=049dcda6920441c5b79957f53025e3f8). Furthermore, the work was developed based on the methodological structure proposed by Arksey & O’Malley (2005) [18], following its expansion [19,20], and adhering to the following stages: 1) defining the research question; 2) aligning the review’s inclusion and exclusion criteria; 3) approaching study selection; 4) selecting, extracting, and presenting evidence; 5) analyzing extracted data; 6) presenting results; and 7) synthesizing data according to the objective.

### Research question

This study aims to answer and discuss the primary applications of aromatherapy with chamomile essential oil in hospital settings for anxiety and pain control. To this end, the question was developed using the PCC mnemonic (Population, Concept, and Context) in accordance with JBI recommendations. Thus, the Population (P) was defined as hospitalized or admitted patients, the Concept (C) as the use of aromatherapy with chamomile essential oil for pain and anxiety management, and the Context (C) as hospital environments. Therefore, the elaborated question was: “What is the effect of using chamomile aromatherapy in pain and anxiety management for patients in hospital settings?”

### Eligibility criteria

For inclusion criteria, the review will consider publications that address the study’s objective, are available in full, and are electronically accessible (via CAFe access); dissertations, theses, ministerial ordinances, guidelines, and scientific articles will be included without temporal restriction and without language exclusion. Abstracts, letters to the editor, opinion articles, off-topic studies, and duplicate records in data sources will be excluded.

### Search strategy

In the first stage, the most appropriate indexing terms and keywords for database searching were selected through consultation with the Health Sciences Descriptors (DeCS) and Medical Subject Headings (MeSH). The chosen descriptors were “Pacientes/Patients,” “Aromaterapia/Aromatherapy,” and “Hospitais/Hospitals,” combined with the Boolean operator “AND” to cross-reference the descriptors. An initial screening by title and abstract will then be performed.

In the second stage, a comprehensive search will be conducted across ten data sources for this review: Medical Literature Analysis and Retrieval System Online (MEDLine/PubMed), Cumulative Index to Nursing and Allied Health Literature (CINAHL), Web of Science, LILACS, Scielo, Cochrane Library, Scopus, ScienceDirect, Wiley Online Library, and Virtual Health Library (VHL). The search will be performed via the CAPES Journal Portal, using remote access through the Federated Academic Community (CAFe) platform. As an example of the search strategy the following string was created for the PubMed database, represented in S2 Table (Table 1), resulting in 168 free full-text documents.

**Table 1:** Example of search strategy in the PubMed database.

Database	Search strategy
Pubmed	((“patient s”[All Fields] OR “patients”[MeSH Terms] OR “patients”[All Fields] OR “patient”[All Fields] OR “patients s”[All Fields]) AND (“aromatherapy”[MeSH Terms] OR “aromatherapy”[All Fields] OR “aromatherapies”[All Fields]) AND (“hospital s”[All Fields] OR “hospitalisation”[All Fields] OR “hospitalization”[MeSH Terms] OR “hospitalization”[All Fields] OR “hospitalised”[All Fields] OR “hospitalising”[All Fields] OR “hospitality”[All Fields] OR “hospitalisations”[All Fields] OR “hospitalizations”[All Fields] OR “hospitalize”[All Fields] OR “hospitalized”[All Fields] OR “hospitalizing”[All Fields] OR “hospitals”[MeSH Terms] OR “hospitals”[All Fields] OR “hospital”[All Fields]))

Source: Authors, 2025.

This search will preferentially be conducted in November 2025, and all articles produced and published up to that point will be included. The subsequent steps are anticipated to be finalized by the end of January 2026.

### Study selection

Studies retrieved from the database search will be imported into the Rayyan platform (https://www.rayyan.ai) for identification and removal of duplicates. Following this, titles and abstracts of all eligible articles will be screened to verify their adherence to the review’s inclusion criteria. At this initial stage, the aim is to be more inclusive; articles will only be excluded if it’s clearly evident that they do not meet the eligibility criteria. Subsequently, after selecting full-text articles, those studies that address the research question will be included in the final sample.

The search results will be fully reported in the final scoping review and presented in a PRISMA-ScR flowchart.

### Data extraction, analysis, and synthesis

Data from the included studies will be extracted using a standardized Excel spreadsheet developed by the authors with the variables better detailed in the next topic of this protocol. Two reviewers will independently perform the data extraction, and any disagreements will be resolved through discussion or consultation with a third reviewer to ensure methodological rigor and minimize bias.

The analysis will be conducted descriptively and thematically, allowing the identification of patterns and variations across the included studies. The extracted data will be organized and synthesized according to the objectives of this scoping review, following the recommendations of the Joanna Briggs Institute (JBI).

The results will be presented in tables and figures to ensure clarity and transparency of reporting. To enhance the analytical depth and ensure comprehensive interpretation, the PAGER framework (Patterns, Advances, Gaps, Evidence for practice, and Recommendations) will be employed. This framework will guide the synthesis and discussion of findings, allowing the results to be structured around recurrent themes, advances in knowledge, persisting limitations, and implications for clinical practice and future research.

## Results

The study selection process will be detailed using the PRISMA flowchart (S1 Fig). Data will be extracted from the included studies based on key characteristics defined by the authors, such as: author(s), year of publication, and country; study design and objective(s); setting (e.g., hospital, ICU, ward, surgical center); and key outcomes. An initial draft of this form is presented in Table 2. This form may be refined as the scoping review progresses. Corresponding authors of included studies will be contacted if necessary to obtain any missing information.

**Table 2:** Data extraction form.

**Author(s)**	**Year of publication**	**Country**	**Study design**	**Objective**	**Setting**	**Outcomes**

Additionally, outcome tables will be used to present the previously mentioned characteristics. The PAGER methodology [21] will be employed to enhance reporting quality and provide greater methodological rigor to the scoping review. PAGER is a tool used to analyze reviews through the following elements: Patterns, Advances, Gaps, Evidence for practice, and Recommendations for future research, as shown in Table 3.

**Table 3:** PAGER methodology.

Patterns	Advances	Gaps	Evidence for practice	Recommendations

## Discussion

Traditional, complementary, and integrative medicines encompass a body of knowledge, practice, and skills rooted in the experiences, beliefs, and theories of diverse cultures. They are employed for health maintenance, as well as for the prevention, diagnosis, improvement, or treatment of physical or mental ailments. These therapies are considered complementary when used alongside conventional medicine but are termed alternative if they replace conventional medical treatments [1,4,22,23].

Consequently, ICHP are increasingly gaining traction in scientific studies. They emerge as safe, accessible, and easily applicable alternatives or complementary practices to standard treatments. This review will provide detailed discussions, based on available literature, regarding aromatherapy as a complementary or alternative therapy and its primary applications in health promotion, prevention, recovery, or rehabilitation [24–26].

A clinical study by Najafi et al. (2017) [27] investigated the effect of aromatherapy with chamomile essential oil on postoperative pain after elective cesarean section under spinal anesthesia, finding it reduced pain and analgesic requirements. Similar findings emerged in a systematic review by Mohammadi and Abdollahzadeh (2025) [28], who analyzed randomized clinical trials on the use of chamomile in the same context and concluded that it has a mild but promising analgesic effect in the postoperative period. This suggests that, although its analgesic effects are modest, it has potential as a complementary approach to conventional pain management. Its mechanism of action is linked to the inhibition of the cyclooxygenase and lipoxygenase pathways, blocking the production of prostaglandins and leukotrienes, mediators directly involved in pain.

Furthermore, the clinical trial included in the systematic review by Mohammadi and Abdollahzadeh (2025) [28] showed that in all interventions using chamomile aromatherapy, no adverse effects were reported. This is consistent with other study [29], which concluded that chamomile is generally safe when used in controlled doses, with self-limiting minor adverse events. The studies also reveal that inhalation appears to be the most effective route of administration compared to topical application for postoperative pain relief.

Regarding anxiety, a systematic review [30], which aimed to explore the effectiveness of aromatherapy in relieving hospitalization-related anxiety in patients with acute myocardial infarction, revealed that inhalation of chamomile essential oil had a significant effect in reducing anxiety, leading the authors to encourage its use in hospital settings by healthcare professionals. Studies indicate that its beneficial effects are related to its ability to bind to benzodiazepine and GABA receptors, which play a crucial role in regulating sleep–wake cycles and reducing neural excitability, thereby promoting sedative and anxiolytic effects [30,31].

In support of this finding, a randomized clinical trial conducted in a cardiology department in Iran and published in 2024 showed that the anxiety score, as well as systolic blood pressure (SBP), diastolic blood pressure (DBP), and heart rate, were lower in the group that used aromatherapy with German chamomile (*Matricaria chamomilla*) compared to the control group. This demonstrates its potential not only to reduce anxiety but also to stabilize hemodynamic parameters [32].

Despite the advances observed, there is still a clear need to consolidate and systematize scientific knowledge on the use of aromatherapy with chamomile essential oil in the hospital setting, especially for pain and anxiety management. The literature points to promising but preliminary results, indicating the importance of more clearly outlining the conditions of use, clinical scenarios, and therapeutic protocols. Therefore, the proposed scoping review seeks to fill this gap by mapping the available evidence and defining the conceptual and practical parameters that guide the clinical application of this therapy. The results are expected to contribute to strengthening the theoretical and empirical basis of ICHP, expanding the understanding of its complementary role to conventional medicine, and providing useful support for healthcare professionals in implementing safe, accessible, and evidence-based integrative approaches.

## Conclusions

Based on current evidence, aromatherapy with chamomile essential oil has shown promising results in managing pain and anxiety in hospital settings. Studies indicate mild but significant analgesic effects and consistent benefits in reducing anxiety, especially when administered via inhalation. Furthermore, research highlights chamomile’s safety profile, with no significant adverse events reported, reinforcing its potential as a complementary practice to conventional medicine.

This scoping review intends to synthesize and systematize existing knowledge about this approach, identifying gaps, trends, and opportunities for clinical application. The results are expected to provide theoretical and practical support for the safe and informed incorporation of chamomile aromatherapy into healthcare, contributing to the strengthening of integrative and complementary practices and to more humanized, accessible, and patient-centered care.

## Supporting information

S1 Fig Study selection flow diagram (adapted from the *Preferred Reporting Items for Systematic Reviews and Meta-Analyses extension for Scoping Reviews*, PRISMA-ScR). (TIFF)

S2 Table Example of search strategy in the PubMed database. (DOCX)

S3 File PRISMA-P Checklist. (DOCX)
