# Supplementary figures and images for: Effect of aromatherapy with Matricaria chamomilla in pain and anxiety management in hospital settings: A scoping review protocol
Source: PLoS One. 2026 Jan 5;21(1):e0339953. doi: 10.1371/journal.pone.0339953 (PMC12768350; doi:10.1371/journal.pone.0339953)

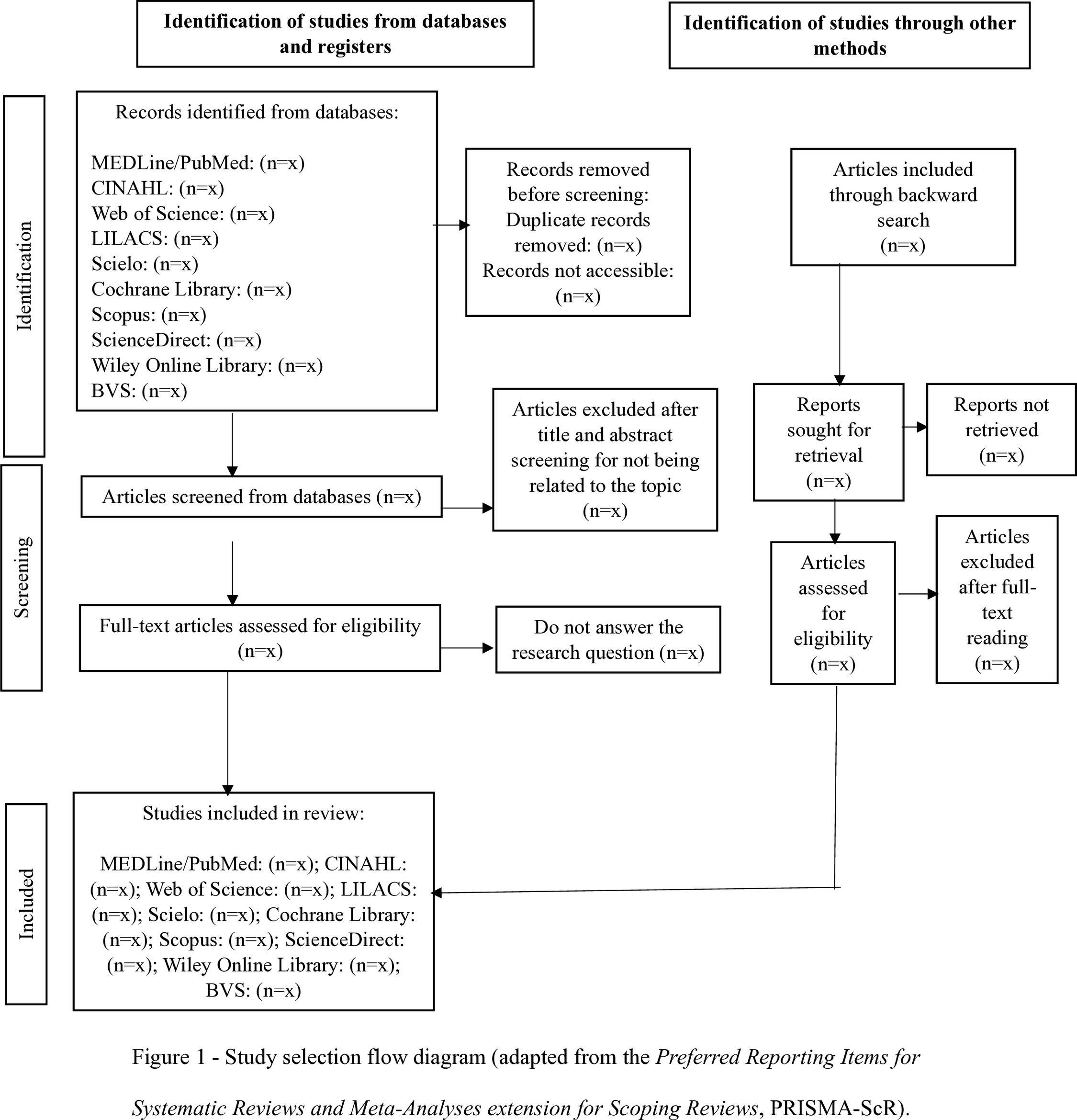

Supplement: S1 Fig — (TIFF) [file pone.0339953.s001.tiff]
