# Supplementary material for: Effect of aromatherapy with Matricaria chamomilla in pain and anxiety management in hospital settings: A scoping review protocol
Source: PLoS One. 2026 Jan 5;21(1):e0339953. doi: 10.1371/journal.pone.0339953 (PMC12768350; doi:10.1371/journal.pone.0339953)
Supplement: S2 Table — (DOCX) [file pone.0339953.s002.docx]

**Table 1. Example of search strategy in the PubMed database.**

| **Database** | **Search strategy** |
| --- | --- |
| Pubmed | (("patient s"[All Fields] OR "patients"[MeSH Terms] OR "patients"[All Fields] OR "patient"[All Fields] OR "patients s"[All Fields]) AND ("aromatherapy"[MeSH Terms] OR "aromatherapy"[All Fields] OR "aromatherapies"[All Fields]) AND ("hospital s"[All Fields] OR "hospitalisation"[All Fields] OR "hospitalization"[MeSH Terms] OR "hospitalization"[All Fields] OR "hospitalised"[All Fields] OR "hospitalising"[All Fields] OR "hospitality"[All Fields] OR "hospitalisations"[All Fields] OR "hospitalizations"[All Fields] OR "hospitalize"[All Fields] OR "hospitalized"[All Fields] OR "hospitalizing"[All Fields] OR "hospitals"[MeSH Terms] OR "hospitals"[All Fields] OR "hospital"[All Fields])) |

Source: Authors, 2025.
